# A nanoparticle therapeutic vaccine targeting HAAH stimulates cellular immunity

**DOI:** 10.1186/2051-1426-3-S2-P442

**Published:** 2015-11-04

**Authors:** Michael Lebowitz, Solomon Stewart, Susan Walker, Samindhi Wu, Steven Fuller, Hossein Ghanbari

**Affiliations:** 1 Panacea Pharmaceuticals, Inc., Gaithersburg, MD, USA

## Background

We are evaluating the immunogenicity and efficacy of a nanoparticle vaccine (NPV) targeting the tumor marker human aspartyl (asparaginyl) β-hydroxylase (HAAH). HAAH is an embryonic protein that is over-expressed on the surface of cancer cells, is demonstrated to be responsible for cell proliferation, motility and invasiveness, processes which can be inhibited by anti-HAAH antibodies in vitro. We have developed novel anticancer NPVs in which portions of the HAAH molecule are expressed on λ-phage (200-300 copies per NPV). These NPVs are immunogenic; producing high-titer anti-HAAH polyclonal antibodies in mice despite the fact that the HAAH protein is highly conserved between mammalian species. We have further shown that the NPVs inhibit tumor growth and metastasis and extends survival in mouse models of liver and breast cancer and in a rat model of prostate cancer. It is generally understood that for a vaccine to provide lasting protective immunity it must also elicit strong cellular immune responses. Here we demonstrate that these HAAH-targeted NPVs can induce antigen specific cytotoxic T cell (CTL) responses.

## Methods

Male Sprague-Dawley rats (N=6/group) were immunized with 2.5x10^11^ phage particles displaying either the N-terminal third (HAAH-1λ) or the C-terminal third (HAAH-3λ) of the HAAH protein. Intramuscular (IM) immunization of the NPV was compared with intradermal (ID) administration using the 3M hMTS device. Vaccinations occurred on Days 0, 14 and 28 and animals were sacrificed on Day 35. Spleens were harvested and CD8^+^ T cells as well as MHC class II^+^ antigen presenting cells (APC) were isolated using magnetic assisted cell sorting (MACS, Milltenyi Biotec). CTL responses were measured using a fluorescent assay in which target cells were pre-loaded with 5-(6)-carboxyfluorescein diacetate succinimidyl ester (CFSE) and dead cells were labelled after incubation with effector cells with 7-amino actinomycin D (7-AAD).

## Results

Antigen specific cytotoxic T cell responses were detected in all vaccinated groups. Specific lysis occurred in HAAH-loaded vs. not loaded APCs derived from the same animals. Specific lysis of HAAH-loaded rat prostate cancer cells (MLLB cells) was also detected. At effector to target ratios of 25:1, specific lysis ranged from 6-28% (Average = 17%) dependent on the vaccine type (HAAH-1 vs. HAAH-3) and the route of delivery (IM vs. ID).

## Conclusions

We have previously demonstrated that HAAH-targeted nanoparticle vaccines are immunogenic and protect against tumor growth and metastasis *in vivo* and extend survival. This work demonstrates that these vaccines also elicit a strong humoral response including development of HAAH-specific cytotoxic T-lymphocyte responses.

